# Maternal Obesity Alters Placental Cell Cycle Regulators in the First Trimester of Human Pregnancy: New Insights for BRCA1

**DOI:** 10.3390/ijms21020468

**Published:** 2020-01-11

**Authors:** Denise Hoch, Martina Bachbauer, Caroline Pöchlauer, Francisco Algaba-Chueca, Veronika Tandl, Boris Novakovic, Ana Megia, Martin Gauster, Richard Saffery, Andreas Glasner, Gernot Desoye, Alejandro Majali-Martinez

**Affiliations:** 1 Department of Obstetrics and Gynecology, Medical University of Graz, 8036 Graz, Austria; denise.hoch@medunigraz.at (D.H.); martina.bachbauer@gmail.com (M.B.); caroline.poechlauer@gmx.at (C.P.); veronika.tandl@stud.medunigraz.at (V.T.); a.majali-martinez@medunigraz.at (A.M.-M.); 2 Department of Endocrinology and Nutrition Research Unit, University Hospital of Tarragona Joan XXIII-Institut d´Investigació Sanitària Pere Virgili (IISPV), 43005 Tarragona, Spain; falgabachueca@gmail.com (F.A.-C.); ana.megia@gmail.com (A.M.); 3 Murdoch Children’s Research Institute, Royal Children’s Hospital, 3052 Melbourne, Australia; boris.novakovic@mcri.edu.au (B.N.); richard.saffery@mcri.edu.au (R.S.); 4 Division of Cell Biology, Histology and Embryology, Gottfried Schatz Research Centre for Cell Signaling, Metabolism and Ageing, Medical University of Graz, 8036 Graz, Austria; martin.gauster@medunigraz.at; 5 Femina Med Center, 8010 Graz, Austria; office@dr-glasner.at

**Keywords:** human placenta, first trimester, early pregnancy, obesity, BRCA1, cell cycle

## Abstract

In the first trimester of pregnancy, placental development involves a wide range of cellular processes. These include trophoblast proliferation, fusion, and differentiation, which are dependent on tight cell cycle control. The intrauterine environment affects placental development, which also includes the trophoblast cell cycle. In this work, we focus on maternal obesity to assess whether an altered intrauterine milieu modulates expression and protein levels of placental cell cycle regulators in early human pregnancy. For this purpose, we use first trimester placental tissue from lean and obese women (gestational week 5^+0^–11^+6^, *n* = 58). Using a PCR panel, a cell cycle protein array, and STRING database analysis, we identify a network of cell cycle regulators increased by maternal obesity in which breast cancer 1 (BRCA1) is a central player. Immunostaining localizes BRCA1 predominantly to the villous and the extravillous cytotrophoblast. Obesity-driven BRCA1 upregulation is not able to be explained by DNA methylation (EPIC array) or by short-term treatment of chorionic villous explants at 2.5% oxygen with tumor necrosis factor α (TNF-α) (50 mg/mL), leptin (100 mg/mL), interleukin 6 (IL-6) (100 mg/mL), or high glucose (25 nM). Oxygen tension rises during the first trimester, but this change in vitro has no effect on BRCA1 (2.5% and 6.5% O_2_). We conclude that maternal obesity affects placental cell cycle regulation and speculate this may alter placental development.

## 1. Introduction

Adequate human placental development is crucial for embryonic and fetal growth. During the first trimester of pregnancy, placental development relies on several cellular processes, including trophoblast proliferation, differentiation, and fusion [1,2]. Within placental villi, the structural unit of the first trimester placenta, villous cytotrophoblasts (vCTs) proliferate and fuse to give rise to the syncytiotrophoblast (ST), a multinucleated cell layer with transport, endocrine, barrier, and protective functions. vCT progenitors can lose their proliferative potential and differentiate into extravillous cytotrophoblasts (EVTs), an invasive cell type that migrates and invades the decidua, thus anchoring the fetoplacental unit to the uterus [3]. EVTs also reach and remodel the maternal spiral arteries to ensure adequate blood supply to the fetus [4,5].

Villous trophoblast turnover and EVT invasion are tightly regulated both in a spatial- and time-dependent manner [6]. Although this regulation has an autocrine and paracrine component mediated by placenta-derived signals, including progesterone, human chorionic gonadotropin, placental lactogen, transforming growth factor β, and kisspeptins, among others [7,8], the maternal intrauterine environment also plays a pivotal role [9]. We have previously described how several factors found in the maternal circulation, such as insulin, tumor necrosis factor α (TNF-α), and endothelin-1, regulate diverse aspects of first trimester trophoblast biology [10,11,12]. Oxygen tension, which rises during the first trimester of pregnancy due to spiral artery remodeling ultimately resulting in flow onset of fully oxygenated blood, is also a major regulator of trophoblast biology [13,14]. It has become increasingly appreciated that maternal environment derangements affecting trophoblast biology during the first trimester of pregnancy may determine trajectories of placental growth, development, and function throughout pregnancy, with ensuing perinatal and long-term consequences for offspring health due to fetal programming [15].

Maternal obesity is characterized by a sustained low-grade pro-inflammatory and metabolically altered environment and entails long term consequences for mother and offspring [16]. Obesity has drastically increased among women of reproductive age during the last decade [17]. Pregnancy complications such as preeclampsia and gestational diabetes mellitus are more common among obese pregnant women, suggesting that maternal obesity affects placental function [18,19]. Additionally, the fetus is affected by maternal obesity, as reflected by the altered fetal metabolome in maternal obesity. This may contribute to the higher incidence of type 2 diabetes, hypertension, and obesity among obese women’s offspring [20].

While obesity-associated adverse effects on maternal and fetal health have been studied in depth at term of pregnancy [21], the effect of maternal obesity on the human first trimester placenta, and specifically on the trophoblast, remains poorly understood [15]. Recent studies have suggested that a tight control of cell cycle progression and cell cycle arrest is required during trophoblast proliferation, fusion, and invasion [22]. Interestingly, maternal obesity has been shown to alter trophoblast transcriptome, affecting genes involved in cell metabolism and cell function [23]. Hence, as part of a major effort to understand effects of maternal obesity on first trimester growth and development, we focus in this study on first trimester placental cell cycle regulation.

Although the expression of classical cell cycle regulators, e.g., cyclin A, B, and D, and Ki67, p21, and p27, has already been described in human first trimester placenta [24,25], for the first time we identify breast cancer 1 (BRCA1) at the core of a network of cell cycle regulators affected by maternal obesity in early pregnancy. We assess its specific location as well as its expression levels in human first trimester placental tissue from lean (gestational week 5^+0^–11^+6^; *n* = 37) and obese (gestational week 5^+0^–11^+2^; *n* = 21) women. Finally, we determine its specific short-term regulation in vitro by obesity-associated pro-inflammatory cytokines and oxygen tension using human chorionic first trimester explants.

## 2. Results

In order to determine the effect of maternal obesity on placental cell cycle modulators, first trimester placental tissue from non-smoking women was divided into two groups, i.e., lean (*n* = 37, mean body mass index (BMI) = 22.2 kg/m^2^) and obese (*n* = 21, mean BMI = 32.3 kg/m^2^). Table 1 shows the sample characteristics for each experimental approach.

### 2.1. Maternal Obesity Affects Placental Cell Cycle Regulators Already in the First Trimester of Pregnancy

A PCR panel and a protein array were used to first test the effect of maternal obesity on gene expression and protein levels of several cell cycle regulators in placental tissue from lean and obese women (week 7). The results were analyzed using a multivariate linear regression (MVLR) model with BMI as the exposure variable adjusting for maternal age.

**Figure 1:**
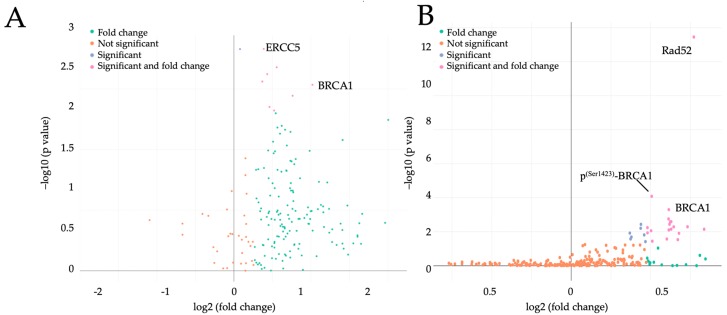
Induction of negative cell cycle regulators by maternal obesity in early pregnancy. Volcano plots show fold change for genes (**A**) and proteins (**B**) differentially expressed between lean (*n* = 7) and obese (*n* = 6) placental tissue (gestational week 7). PCR panel and protein array results were analyzed through multivariate linear regression using BMI as exposure, adjusting for maternal age. Differences were considered significant when *p* < 0.05 and the fold change threshold was set to 1.3. *x* axis = log2 fold change (lean versus obese), *y* axis = multivariate linear regression *p* value. Legend: BRCA1, breast cancer 1; ERRC5, excision repair 5; p^(Ser1423)^-BRCA1, phospho-BRCA1.

Only cell cycle regulators with a fold change (FC) > 1.3 and *p* < 0.05 were considered significant. Maternal obesity increased the expression of 9 out of 187 (4.8%) cell cycle regulators (Supplementary Table S1), with excision repair 5 (*ERCC5*) and *BRCA1* showing the highest fold change between lean and obese (1.5-fold, *p* = 0.02 and 2.0-fold, *p =* 0.03, Figure 1A, respectively). Among the cell cycle regulating proteins, 25 out of 95 (26.3%) were also increased (Supplementary Table S2), with Rad52 and BRCA1 showing the highest fold change (1.5-fold, *p* < 0.0001 and 1.4-fold, *p =* 0.002, respectively, Figure 1B). Phospho-BRCA1 (p^(Ser1423)^-BRCA1) was also significantly upregulated by maternal obesity (FC = 1.3, *p =* 0.009, Figure 1B).

### 2.2. BRCA1 Is a Key Player in Cell Cycle Regulation in Early Pregnancy and Is Upregulated by Maternal Obesity

**Figure 2:**
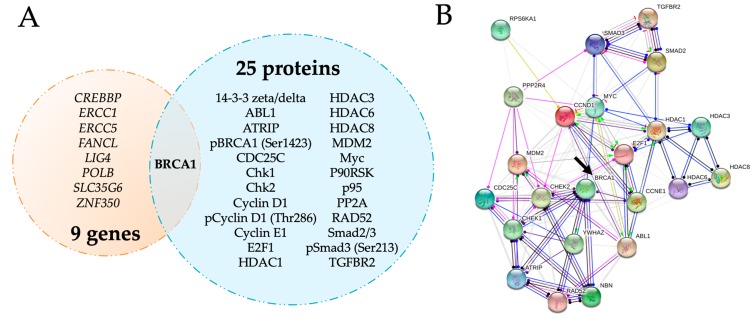
Central role of placental BRCA1 in the first trimester of pregnancy in the context of maternal obesity. Venn diagram depicting cell cycle genes and proteins regulated by maternal obesity (BMI ≥ 30 kg/m^2^) in the first trimester of pregnancy with BRCA1 as the only common factor (**A**). Protein–protein interaction analysis of the upregulated proteins using the STRING database shows a central role of BRCA1 in the cell cycle regulation network (**B**, arrow). Line shape indicates the predicted mode of action, with nodes describing protein action effects (arrow: positive, dash: negative, circle: unspecified) and line color describing protein action types (blue: binding, black: reaction, green: activation, red: inhibition, pink: posttranslational modification, violet: catalysis). The minimum required interaction score was set to medium confidence (0.4), showing up to 10 interactions in the first shell.

Among all the cell cycle regulators analyzed in the present study, only BRCA1 mRNA and protein were concordantly upregulated by maternal obesity (Figure 2A and Supplementary Figure S1). Using the STRING database network analysis tool, we established a protein–protein association network to identify possible functional interactions between these obesity-upregulated cell cycle modulators. Within this network, BRCA1 was located at a central position (Figure 2B, arrow), interacting with proteins involved in different cell cycle events such as Chk1, Chk2, and Myc.

**Figure 3:**
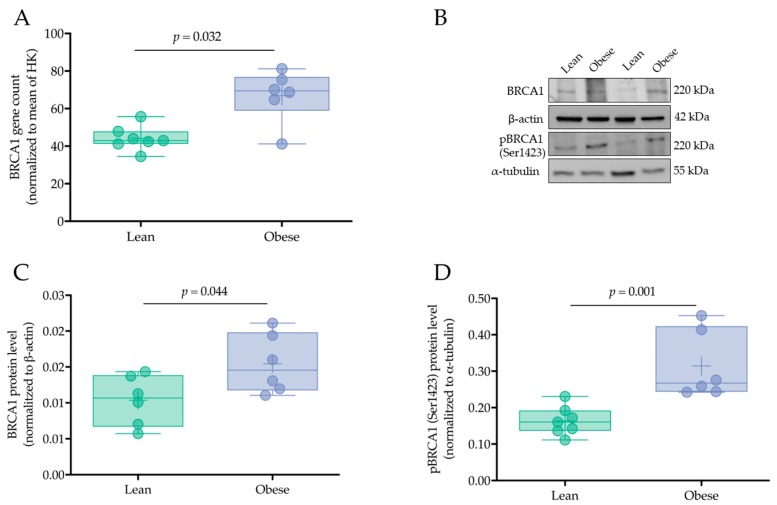
Placental BRCA1 is upregulated by maternal obesity. BRCA1 expression and protein levels were determined in first trimester placental tissue (gestational week 7) from lean (*n* = 6–7) and obese (*n* = 6) women by Nanostring (**A**) and Western blotting (**B**–**D**), respectively. Gene counts of Nanostring analysis were normalized to the mean of two different housekeeping (HK) genes (WD repeat domain 45B (*WDR45L*) and TATA box binding protein (*TBP*, **A**). Immunoblots for BRCA1 and p^(Ser1423)^-BRCA1 (**B**) were quantified by densitometric analysis (**C** and **D**). β-actin and α-tubulin were used for normalization as loading controls. Results are presented as mean ±  SD. Statistical analysis was performed using the Mann Whitney test or *t*-test as appropriate.

BRCA1 upregulation by maternal obesity was confirmed by Nanostring analysis and Western blotting. Nanostring data showed an increase in *BRCA1* expression by 52.3% in placental tissue from obese versus lean women (*p =* 0.032, Figure 3A). Similarly, placental BRCA1 and p^(Ser1423)^-BRCA1 protein levels were also increased within the obese cohort (by 49.1%, *p* = 0.044 and 57.4%, *p* = 0.001, respectively) compared to the lean cohort (Figure 3B–D). The differences observed were independent of fetal sex.

### 2.3. BRCA1 DNA Methylation Is Not Altered by Maternal Obesity

**Figure 4:**
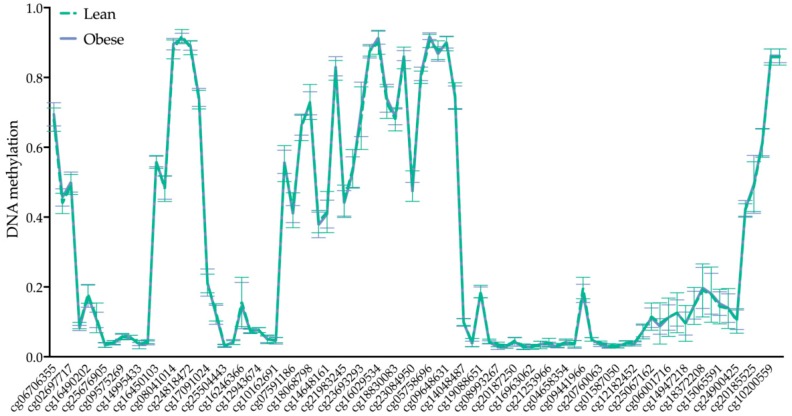
DNA methylation profile of *BRCA1* gene is not altered by maternal obesity in the first trimester of pregnancy (*n*_(lean/obese)_ = 15/15). Genome-wide DNA methylation quantification using Infinium MethylationEPIC Array (850 K) BeadChip included 86 CpGs annotated to the *BRCA1* gene.

Obesity influences placental gene expression through DNA methylation [26]. To test whether obesity-associated BRCA1 upregulation is the result of epigenetic changes in early pregnancy, placental BRCA1 methylation was assessed. We found no evidence that maternal obesity alters the DNA methylation profile of the 86 CpGs in the *BRCA1 g*ene in the first trimester placenta (Figure 4). Mean beta values for the sum of all CpG sites of obese total tissue DNA samples (0.319, gestational week 6^+0^–11^+6^, *n* = 15) were similar to those of the lean group (0.318, gestational week 5^+0^–11^+2^, *n* = 15). The BRCA1 methylation profile was also not affected by maternal age (data not shown). A list with all analyzed CpGs can be found in Supplementary Table S3.

### 2.4. Placental BRCA1 Is Mainly Localized to vCTs and EVTs during the First Trimester of Pregnancy

**Figure 5:**
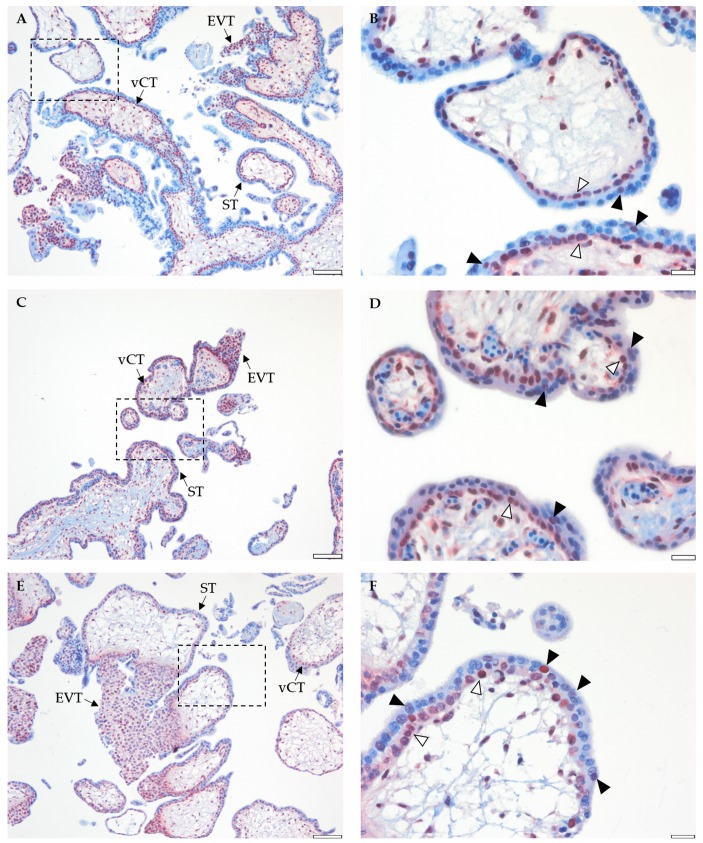
Immunohistochemistry of placental tissue from early (gestational week 5, **A** and **B**), mid (gestational week 8, **C** and **D**), and late (gestational week 12, **E** and **F**) first trimester localizing BRCA1 to the nuclei and the cytoplasm of villous cytotrophoblasts (vCTs, **A**, **C**, and **E**) and extravillous cytotrophoblasts (EVTs, **A**, **C**, and **E**). Syncytiotrophoblast (ST) BRCA1 immunostaining was located in the nuclei (**B**, **D**, and **F,** arrowheads. Open arrowheads indicate vCTs). Scale bar: 100 µm (**A**, **C** and **E**) or 20 µm (**B**, **D** and **F**). Dotted frames in **A**, **C**, and **E** indicate those fields shown with a higher magnification in **B**, **D** and **F**. Positive (ovarian cancer specimen) and negative (IgG isotype) controls (Supplementary Figure S2).

BRCA1 location in early, mid, and late first trimester placental tissue (gestational week 5, 8, and 12, respectively) was detected by immunostaining. Both vCTs and EVTs showed strong BRCA1 staining which was localized to the nuclei and the cytosol (Figure 5A, C, E). Stromal cells were also stained with anti-BRCA1. BRCA1 immunostaining in the ST was located in the nuclei (Figure 5B, D, F, arrowheads). No differences in location were observed between early, mid, and late first trimester placental specimens. Negative (immunoglobulin G isotype) and positive (ovarian tumor section) controls showed no signal or strong nuclear and cytosolic BRCA1 staining, respectively (Supplementary Figure S2).

### 2.5. Short Term Exposure to Obesity-Associated Cytokines, Hyperglycemia, and Increased Oxygen Tension Do Not Regulate Placental BRCA1 Levels in Early Pregnancy

**Figure 6:**
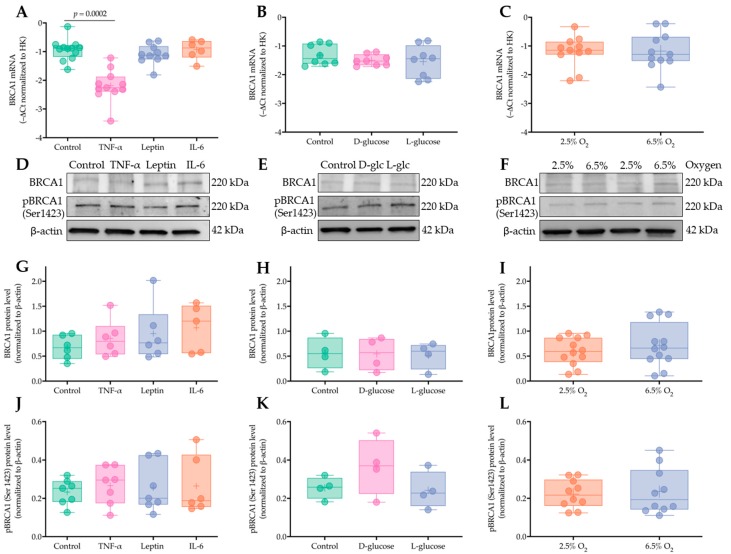
BRCA1 is not regulated by short term exposure to obesity-associated inflammation, hyperglycemia, or oxygen tension in early pregnancy. First trimester placental chorionic villous explants from different placental tissues (*n* = 4–11, gestational week 5–11) were cultured at 2.5% O_2_ with tumor necrosis factor α (TNF-α) (50 ng/mL), leptin (100 ng/mL), interleukin 6 (IL-6) (100 ng/mL), D-glucose (25 nM), and L-glucose (25 nM, osmotic control) for 48 h in triplicate. Explants (*n* = 10–12) were also cultured at 6.5% O_2_. *BRCA1* gene expression was analyzed by RT-qPCR and normalized to the mean of hypoxanthine phosphoribosyltransferase 1 (*HPRT1*) and peptidylprolyl isomerase A (*PPIA*), which were used as housekeeping (HK) genes (**A**-**C**). BRCA1 and p^(Ser1423)^-BRCA1 protein levels were analyzed by Western blotting (**D**–**F**). Immunoblots were quantified by densitometric analysis (**G**–**I** for BRCA1 and **J**–**L** for p^(Ser1423)^-BRCA1), with β-actin used as a loading control. Data are shown as –ΔCt (**A**–**C**) or ratio to β-actin (**G**–**L**) and have been presented as mean ± SD from different placental tissues (*n* = 4–12). Statistical analysis included the Mann Whitney test or Friedman’s test followed by Dunn’s post hoc analysis.

To identify whether potential components of the pro-inflammatory environment associated with obesity can drive acute BRCA1 upregulation in the first trimester placenta, human chorionic villous explants (gestational week 5^+0^–10^+4^, *n* = 6–11) were incubated with TNF-α (50 ng/mL), leptin (100 ng/mL), and interleukin 6 (IL-6) (100 ng/mL) for 48 h. *BRCA1* expression was determined by RT-qPCR (Figure 6A) and BRCA1 and p^(Ser1423)^-BRCA1 protein levels were measured by Western blotting (Figure 6D, G, J). TNF-α induced a downregulation of *BRCA1* expression (−42% versus control, *p* < 0.001). However, BRCA1 and p^(Ser1423)^-BRCA1 protein levels remained unaffected. No effects were found upon leptin and IL-6 treatment.

In a similar experimental set-up, we investigated the influence of hyperglycemia on BRCA1. Chorionic villi (gestational week 6^+4^–10^+4^, *n* = 4–8) were incubated with high glucose (D-glucose, 25 nM) and L-glucose (25 nM) used as an osmotic control. High glucose did not affect BRCA1 mRNA (Figure 6B) or protein levels (Figure 6E, H). BRCA1 Ser1423-phosphorylation was also not altered by glucose treatment (Figure 6E, K).

All the experiments were performed at 2.5% O_2_, which was considered as a physiological O_2_ tension for early first trimester placenta. Since oxygen tension in the intervillous space rises during the first trimester period covered by the explant experiments (weeks 5–10), we tested the potential effect of oxygen on BRCA1. To this end, chorionic villi (gestational week 5^+0^–9^+1^, *n* = 10–12) were also cultured under 6.5% O_2_ and the results compared to 2.5% O_2_. No differences in *BRCA1* expression (Figure 6C) and BRCA1 and p^(Ser1423)^-BRCA1 protein levels (Figure 6F, I, L) were detected between the two oxygen tensions.

The ratio between p^(Ser1423)^-BRCA1 and total BRCA1, reflecting activity of upstream ataxia telangiectasia mutated (ATM) kinase, was also not affected by any of the treatments described above (Supplementary Figure S3). In principle, the absence of a response to most of the treatments may have been due to low tissue viability. This can be ruled out since human chorionic gonadotropin, a major hormone produced by the trophoblast, was secreted into the culture medium, and its levels remained stable during the various treatments (Supplementary Figure S4).

## 3. Discussion

Adequate trophoblast proliferation, differentiation, fusion, and survival are required for successful placental development and function [22]. Although cell cycle regulation is crucial for these biological processes [27], placental cell cycle modulators and their potential regulation by the intrauterine environment have been scarcely investigated.

Obesity has been associated with low-grade sustained inflammation and oxidative stress, both classical triggers of cell cycle arrest [28]. In the present study, we demonstrated that maternal obesity increases the expression of several cell cycle regulators, i.e., 9 genes and 25 proteins, in the first trimester of pregnancy, suggesting that obesity affects placental cell cycle control already in early pregnancy. This might in turn compromise vCT proliferation and differentiation into EVTs, altering invasion and spiral artery remodeling, and may, thus, set the basis for obesity-associated pregnancy disorders such as preeclampsia [29]. Advanced maternal age is known to alter placental cell proliferation and apoptosis [30,31] and might also modify cell cycle regulation. Thus, this was accounted for in the present study by adjusting exposure–outcome relationships for maternal age. This should allow for the identification of obesity-mediated effects, although residual confounding cannot be excluded.

To subsequently characterize the effect of maternal obesity on specific cell cycle modulators, we selected BRCA1 as a potential candidate based on its consistent upregulation at the mRNA and the protein level. BRCA1 has been traditionally studied in the context of breast and ovarian cancer [32], where it plays a pivotal role in establishing an adequate DNA damage response [33,34]. It also plays an important role in cell cycle checkpoint regulation, inducing G1 cell arrest through p27 activation, blocking S phase entry through p53-dependent activation, and favoring G2/M arrest through p53-dependent 14-3-3 zeta/delta activation [35,36]. Interestingly, we found that BRCA1 was the only cell cycle modulator upregulated at both the gene and protein level by maternal obesity. Several BRCA1 protein binding partners were also upregulated by maternal obesity, including 14-3-3 zeta/delta, chk1, and chk2, and the latter is known to induce cell cycle arrest in a pathway involving cdc25 and p-BRCA1 [37,38]. We also observed that p^(Ser1423)^-BRCA1 was upregulated by maternal obesity. Several kinases are involved in BRCA1 phosphorylation, including chk2, Akt, and ATM, the latter being directly involved in BRCA1 phosphorylation at serine 1423 [39]. Phosphorylation of BRCA1 fine-tunes its function. The enhanced phosphorylation in obesity might reflect further BRCA1 interactions with other cell cycle regulators [40]. Indeed, our network analysis of functional protein associations using the STRING database identified BRCA1 as central in the pathways controlling placental cell cycle regulation in early pregnancy.

Only a few reports have investigated BRCA1 location in late first trimester and term human placental tissue [41,42]. Thus, a thorough characterization of BRCA1 location in early pregnancy was still required. We localized BRCA1 predominantly to vCTs and EVTs in early, mid, and late first trimester placental tissue. BRCA1 immunostaining within the ST was weaker and restricted to a few nuclei. A similar distribution pattern, i.e., absence or weak expression in ST versus high expression on vCTs and EVTs, has been described for several other cell cycle regulators including Ki67, cyclin A, p53, and p57 [24,43]. Considering that ST is characterized by the absence of an active cell cycle [44], BRCA1 positive nuclei within the ST might be the result of recent vCT–ST fusion.

It has been previously reported that subcellular BRCA1 location also determines its function [45]. We detected BRCA1 in the nuclei and cytosol of vCTs. Cytosolic BRCA1 location has been associated with highly proliferating breast cancer tumors [46]. Its presence in the cytosol of vCTs reflects the high proliferative potential, as can be expected from the trophoblast stem cell population. The essential role of BRCA1 for vCTs is also demonstrated by BRCA1 knock-down leading to increased apoptosis in the first trimester trophoblast cell line Swan71 [42], suggesting that BRCA1 promotes trophoblast survival. Likewise, its presence in EVTs might reflect the role of BRCA1 in cell cycle regulation during the gradual differentiation of vCTs into EVTs, which precedes invasion. Whether BRCA1 is directly involved in regulating cell proliferation and invasion in primary vCTs and EVTs needs to be further studied.

We then aimed to determine which obesity-associated molecular mechanisms could explain the differences observed in placental BRCA1 levels between lean and obese women. Among other mechanisms obesity has been shown to alter gene expression through DNA methylation [47], which may be a candidate mechanism to explain these differences. This hypothesis is also supported by a recent study demonstrating BRCA1 downregulation due to promoter hypermethylation in disorders characterized by trophoblast over-proliferation [48]. Despite the overall effects of obesity on placental DNA methylation [26], placental BRCA1 methylation profile was not affected by maternal obesity in early pregnancy.

To identify short-term drivers of BRCA1 changes related to obesity we chose those cytokines most prominently associated with the pro-inflammatory environment of obesity [49] and tested them in an in vitro villous explant culture model. TNF-α treatment altered *BRCA1* gene expression without concomitant changes of BRCA1 protein. This lack of a short-term effect on BRCA1 protein levels does not preclude the possibility that chronic exposure to TNF-α in vivo can contribute to BRCA1 upregulation, as found in the obese cohort. Similarly, IL-6 and leptin did not regulate BRCA1 protein. Although we carefully selected cytokine concentrations within the physiological range to mimic the in vivo environment in first trimester placenta [50], we did not investigate a potential interplay between these cytokines, which might also fine-tune placental BRCA1 regulation. Moreover, treatment of placental chorionic villous explants from lean women with pro-inflammatory cytokines only allows testing for their short-term effects (up to 48 h), which may not be long enough to induce changes seen as the result of long-term tissue exposure to the obesity environment.

Obesity-associated hyperglycemia has been shown to alter placental development and function [51]. Interestingly, hyperglycemia is able to promote cell cycle arrest via cyclin D1 and p21 upregulation [52]. Here, high glucose treatment did not alter human placental BRCA1 gene expression or protein levels. Intriguingly, women with BRCA1 mutations are more prone to developing diabetes, a risk that also increases in women with a high BMI [53]. In this regard, hyperinsulinemia is also a common feature of both obesity and diabetes, and high insulin levels have been linked to cell cycle arrest in mouse keratinocytes [54]. Thus, further studies assessing the potential role of obesity-associated hyperinsulinemia on BRCA1 levels are warranted.

Oxygen tension is one of the major regulators of vCT proliferation and EVT differentiation. Hence, we determined whether oxygen might directly regulate first trimester placental BRCA1 levels. Physiological oxygen concentrations rise from 2.5% to 6.5% O_2_ in early pregnancy [55]. Although we have shown that BRCA1 and p^(Ser1423)^-BRCA1 levels remained stable under both physiological oxygen concentrations, BRCA1-associated RING domain protein 1 (BARD1) is increased in first trimester trophoblast under physiological low oxygen tension (6.5% O_2_) [56]. The BARD1–BRCA1 dimer results in BRCA1 stabilization [35]. Thus, BRCA1 activity might indirectly be regulated by oxygen tension.

To the best of our knowledge, this is the first study assessing the influence of maternal obesity on placental cell cycle regulators during the first trimester of pregnancy. The results using placental tissue, which preserves the spatial arrangement of cells and the extracellular matrix, in which they are embedded, clearly demonstrated an effect of maternal obesity on several cell cycle modulators. Among these we could identify BRCA1 as a central node in the network of cell cycle regulator proteins. It localizes to vCTs and EVTs independently of maternal BMI. Key components of the obesity-associated inflammatory and metabolic environment are unlikely to contribute to these changes. The physiological increase in oxygen tension and DNA methylation do also not appear to drive BRCA1 upregulation in obesity.

In the present study all the experiments were conducted on human first trimester placental tissue in a physiological, i.e., low, oxygen environment. This is a major strength and avoids potential hyperoxic effects of ambient oxygen (21% O_2_). The main limitation of this study was the lack of an in-depth analysis of the cellular consequences of obesity-associated BRCA1 changes. Moreover, concentration and time-dependent effects of pro-inflammatory cytokines and the hyperglycemic environment on BRCA1 regulation cannot be excluded. Likewise, other maternal metabolic factors that could explain the obesity associated long-term effects on BRCA1 levels, e.g., hyperinsulinemia and insulin resistance, were not investigated in the present study and their contribution cannot be ruled out.

Collectively, our results suggest that BRCA1 might play a role in first trimester trophoblast biology. Thus, its upregulation by maternal obesity might alter placental development with potentially ensuing adverse consequences for maternal and fetal health.

## 4. Materials and Methods

### 4.1. Study Subjects

The study was approved by the institutional review board and the ethical committee of the Medical University of Graz (29-095 ex16/17, 23 December 2016). Women with a singleton pregnancy scheduled for legal elective pregnancy termination were recruited upon signing written informed consent. Since smoking has major effects on metabolism and inflammation, we carefully excluded smoking women, who were identified by serum cotinine levels ≥0.03 nmol/L [57]. Women with other comorbidities and those under current medication were also excluded from the study.

Upon inclusion, clinical data was collected (Table 1). Maternal BMI was calculated using the formula BMI = weight (kg)/height (m)^2^. Gestational age was calculated based on the patient’s last menstrual period and verified by measurement of the fetal crown-rump length.

### 4.2. Human Placental Tissue Collection

First trimester placental tissue (gestational week 5^+0^–11^+6^) was obtained after surgical pregnancy termination, washed with phosphate-buffered saline (PBS) and cryopreserved at −80 °C until further use, or was fixed in 4% paraformaldehyde (PFA) and paraffin-embedded. Fetal sex was assessed by gene expression analysis (*DDX3Y* and *XIST*, see 4.6). Based on the maternal pre-pregnancy BMI, gestational age matched samples were subsequently divided into two groups, i.e., lean (mean BMI = 22.2 kg/m^2^) and obese (mean BMI = 32.3 kg/m^2^).

### 4.3. First Trimester Chorionic Villous Explants

Human first trimester chorionic villi were micro-dissected into small pieces (15–20 mg wet weight), rinsed with PBS, and cultured in Dulbecco’s Modified Eagle Medium (DMEM; Gibco, Invitrogen, Carlsbad, CA, USA) and Ham’s F-12 medium 1:1 (*v*/*v*; Gibco) supplemented with 10% fetal calf serum (FCS, Thermo Scientific, Rockford, IL, USA) and 1% penicillin-streptomycin (Gibco). Placental explants were cultured in a hypoxic workstation (BioSpherix; Redfeld, NY, USA). After 24 h of pre-incubation to allow for adjustment to the in vitro conditions, samples were treated with TNF-α (50 ng/mL, Sigma Aldrich, St. Louis, MO, USA), leptin (100 ng/mL, Sigma Aldrich), IL-6 (100 ng/mL, Sigma Aldrich), D-Glucose (25 mM, Merck, Billerica, MA, USA), or L-Glucose (25 mM, Sigma Aldrich) at 2.5% O_2_ for 48 h. The effect of oxygen tension was additionally assessed at 6.5% O_2_ for 48 h. Thereafter, explants were snap-frozen for subsequent RNA extraction or protein isolation. Culture supernatant was frozen and used for human chorionic gonadotropin (hCG) analysis (see Supplementary Materials).

### 4.4. DNA/RNA Isolation and Reverse Transcription

First trimester placental tissue was homogenized in RLT Plus Buffer (Qiagen, Venlo, Netherlands) with 1% β-mercaptoethanol (*v*/*v*, Merck) using a tissue lyser (MagNa Lyser, Roche, Basel, Switzerland). DNA and total RNA was isolated with the AllPrep DNA/RNA/miRNA Universal Kit (Qiagen) according to the manufacturer’s guidelines. After a quality check (Bioanalyzer, RNA integrity number (RIN) > 3.0), mRNA reverse transcription was performed using the SuperScript II Reverse Transcriptase kit (Life Technologies, Carlsbad, CA, USA) as per the manufacturer’s protocol.

### 4.5. PrimePCR Panel

Differential expression of 187 genes associated with DNA damage-repair and cell cycle regulation was analyzed using a PrimePCR Collection panel (DNA damage H384 Predesigned 384-well, BioRad Laboratories, Munich, Germany) according to the manufacturer´s guidelines. A complete list of the genes and controls can be found on the manufacturer’s website (https://www.bio-rad.com/de-at/prime-pcr-assays/pathway/dna-damage-collection-panel). For each PCR reaction 10 ng of cDNA were used. Real-time PCR was then conducted using the CFX384 PCR detection system (BioRad Laboratories) and Ct values were generated by the associated software. Results were analyzed using the 2^−ΔΔ*C*t^ method. Hypoxanthine phosphoribosyltransferase 1 (*HPRT1*) and TATA box binding protein (*TBP*) were selected as housekeeping genes since their expression was unaffected by maternal obesity.

### 4.6. Real Time PCR

*BRCA1* expression was determined by quantitative real-time PCR using FAM-labeled TaqMan gene expression assays (Life Technologies, *BRCA1*: Hs01556193_m1). Fetal sex was determined in a multiplex PCR setup using FAM-labelled *DDX3Y* and VIC-labelled *XIST* expression assays (Life Technologies, *DDX3Y*: Hs00965254_gH, *XIST*: Hs01079824_m1) as described elsewhere [58]. RT-qPCR was performed using TaqMan Universal PCR Master Mix (Life Technologies) using the CFX96 Thermocycler (BioRad Laboratories). A calibrator sample was added onto each plate to control for inter-run variations. Ct values were generated by the BioRad CFX Manager 3.1 software and relative gene expression was calculated by the 2^−ΔΔ*C*t^ method, with *HPRT1* (Life Technologies, *HPRT1*: Hs02800695_m1) and peptidylprolyl isomerase A (Life Technologies, *PPIA*: Hs04194521_s1) used as housekeeping genes.

### 4.7. Nanostring

PrimePCR Panel validation was performed using the NanoString nCounter system (Nanostring Technologies, Seattle, WA, USA), which is based on direct digital detection of mRNA molecules using target-specific, color-coded probe pairs that hybridize directly to target molecules. Gene expression was measured by counting the barcode for each specific molecule, which is detected by a digital analyzer. Positive normalization to the geo-mean of the top three positive controls and codeset normalization on the reference genes WD repeat domain 45B (*WDR45L*) and *TBP* was performed using nSolver software (Nanostring Technologies). Results have been expressed as gene counts of mRNA molecules in 100 ng/µL RNA.

### 4.8. Protein Isolation and Quantification

Placental tissue was lysed in RIPA buffer (Sigma Aldrich) containing complete protease inhibitors (Roche) using a tissue lyser (MagNa Lyser, Roche). Protein concentration was determined using the bicinchoninic acid assay (BCA, Thermo Fisher Scientific) as per the manufacturer’s guidelines.

### 4.9. Cell Cycle Control Protein Array

Cell cycle-related protein profile was determined using the Cell Cycle Control Phospho Antibody Array (95 site- and phospho-specific antibodies, Fullmoon Biosystems, Sunnyvale, CA, USA) according to the manufacturer´s guidelines. Briefly, slides were treated with blocking solution (Fullmoon Biosystems) for 30 min at room temperature and incubated with 75 μg of biotin-labelled first trimester placental protein lysates overnight at 4 °C. After washing, conjugated proteins were detected using Cy3-conjugated streptavidin. Image analysis was performed using GenePix Pro 7.0 software (Molecular Devices, San Jose, CA, USA). After local background subtraction, data were normalized on the median intensity value of all antibodies on each array (excluding empty spots and negative/positive markers). Only those signals exceeding the background intensity by two-fold were considered.

### 4.10. Immunoblotting

Equal amounts of total protein were mixed with Laemmli-buffer (Sigma) and denatured for 5 min at 96 °C. Ten micrograms of total protein were loaded onto 4–20% SDS-PAGE gels (BioRad Laboratories), resolved for 1 h at 110 V, and transferred to nitrocellulose membranes using the BioRad TurboBlot (BioRad Laboratories). Blotting efficiency was determined with Ponceau staining (Ponceau S solution, Sigma-Aldrich). Membranes were blocked for 1 h with 5 % non-fat dry milk (Bio-Rad) in tris-buffered saline (TBS) and 0.1% Tween 20 (Sigma), and subsequently incubated with anti-BRCA1 (1:1000, Sigma-Aldrich, AB-1423), anti-p^(Ser1423)^-BRCA1 (1:1000, Merck, 07-635), anti-α-tubulin (1:1000, Merck, CP06), and anti-β-actin (1:10000, ab8227, Abcam, Cambridge, UK) antibodies overnight at 4 °C. Thereafter, membranes were incubated with the appropriated horseradish peroxidase (HRP)-conjugated secondary antibody (BioRad Laboratories, 1:2000 for BRCA1, p^(Ser1423)^-BRCA1, and α-tubulin, 1:25,000 for β-actin) for 1 h at room temperature. Immunodetection using SuperSignal-Pico Chemiluminescent Substrate (Thermo Scientific) was visualized with a Fusion FX instrument (Vilber Lourmat, Collégien, France). Band intensity was quantified using EvolutionCapt software (Vilber Lourmat). To account for inter-membrane variation, data was normalized to an internal calibrator sample (first trimester placental tissue) included in every gel.

### 4.11. Immunohistochemistry

BRCA1 immunostaining was performed on 3 µm thick sections of paraffin-embedded first trimester placental tissue. After deparaffinization, antigen retrieval was performed using 10 mM citrate buffer (pH 6) at 110 °C for 10 min in a Decloaking Chamber (Biocare medical, Pacheco, CA, USA). BRCA1 immunohistochemistry was performed using the UltraVision LP Detection System (HRP polymer kit, Thermo Fisher Scientific) as per the manufacturer´s instructions. Briefly, endogenous peroxidase was blocked with UltraVision Hydrogen Peroxide Block for 10 min followed by an incubation with UltraVision Protein Block for 5 min at room temperature. Anti-BRCA1 antibody (1:500, Sigma-Aldrich, AB-1423) was diluted in Antibody Diluent (Dako, Glostrup, Denmark) and incubated for 1 h at room temperature in a humidified chamber. Antibody detection was performed after incubation with Primary Antibody Enhancer using an HRP-labeled polymer (Dako) and 3-amino-9-ethyl-carbazole Single Solution (Thermo Scientific). Nuclei were counterstained with Mayer’s hematoxylin (Gatt Koller, Absam, Austria) and slides were mounted with Aquatex (Merck, Darmstadt, Germany). Images were acquired with a Zeiss Axio Z1 microscope (Zeiss, Oberkochen, Germany) equipped with a digital camera (Olympus X, Tokyo, Japan) using AxioVision Software (Zeiss).

### 4.12. DNA Methylation Profiling

Genomic DNA samples were sent to HuGe-F (Erasmus MC, Rotterdam, Netherlands) for sodium bisulfite treatment and genome-wide methylation analysis using Illumina InfiniumMethylationEPIC BeadChips. Raw data (IDAT files) were exported from GenomeStudio (Illumina, San Diego, CA). The Bioconductor package missMethyl was used to read the data into R and carry out quality control, pre-processing, and normalization using the subset-quantile within array normalization (SWAN) method [59,60]. The Bioconductor limma package was used to fit a linear model to compare lean and obese placental tissue samples.

*M* values were calculated after removing poor performing probes (*p* value cut-off > 0.05 for all samples) and probes on the sex chromosomes. Beta values were derived from intensities as defined by the ratio of methylated to unmethylated probes (β = M/(U + M + 100)) and were used as a measure of effect size.

### 4.13. Statistics

Statistical analysis was performed with GraphPad Prism 8, IBM SPSS Statistics 25, and R 3.6.1 [61]. Normal distribution of the data was determined using the Kolmogórov-Smirnov test. Associations between maternal obesity and gene expression or protein levels were assessed using an MVLR model adjusted for maternal age, since advanced maternal age might also alter placental cell cycle regulation [30,31]. In this model, maternal age was considered a continuous variable and maternal BMI was categorized into lean (mean BMI = 22.2 kg/m^2^) and obese (mean BMI = 32.3 kg/m^2^). Volcano plots were generated using the linear regression *p* value (−log10) and a pre-calculated fold change (log2) and visualized using the *R* Bioconductor packages ggplot2 [62], gridExtra [63], and plotly [64]. *p* < 0.05 together with a fold change exceeding 1.3 was considered statistically significant. Normally distributed data were analyzed using a *t*-test and one-way ANOVA. For non-parametric analysis, Mann Whitney or Friedman’s test followed by Dunn’s post hoc test was used. *p* < 0.05 was considered statistically significant. The STRING database (https://string-db.org/) was used to visualize functional protein interaction and protein array data is presented as a connectivity network showing action effects and types.

## Figures and Tables

**Table 1:** Description of the study cohorts by experiment.

Characteristics	PCR Panel and Protein Array			Methylation			IHC	Explants
	Lean	Obese	*p*	Lean	Obese	*p*	Lean	Lean
Sample size (*n*)	7	6		15	15		4	11
Gestational age (days)	49.0 ± 0.0	49.8 ± 1.8	0.3	57.3 ± 13.5	56.1 ± 13.1	0.7	53.0 ± 17.5	51.9 ± 11.9
Maternal age (years)	33.0 ± 5.8	36.7 ± 5.3	0.3	27.8 ± 7.0	30.3 ± 6.0	0.3	31.7 ± 9	28.2 ± 7.4
Maternal BMI (kg/m^2^)	20.3 ± 1.6	30.3 ± 2.3	<0.0001	22.2 ± 1.6	34.3 ± 3.4	<0.0001	24.5 ± 3.3	21.9 ± 3.3
Fetal sex	4 m; 3 f	3 m; 3 f		6 m; 9 f	9 m; 6 f		1 m; 3 f	6 m; 5 f

Data are expressed as mean ± SD. The Mann-Whitney test was used for statistical analysis. Legend: BMI, body mass index; IHC, immunohistochemistry; m, male; f, female.

## Supplementary Materials

Supplementary materials can be found at https://www.mdpi.com/1422-0067/21/2/468/s1.

## Abbreviations

ATM	Ataxia telangiectasia mutated kinase
BCA	Bicinchoninic acid assay
BMI	Body mass index
*BRCA1*	Breast cancer 1
CRL	Crown-rump length
DMEM	Dulbecco’s Modified Eagle Medium
EVTs	Extravillous trophoblasts
FC	Fold change
hCG	Human chorionic gonadotropin
*HPRT1*	Hypoxanthine phosphoribosyltransferase 1
IHC	Immunohistochemistry
IL-6	Interleukin 6
MVLR	Multivariate linear regression
PFA	Paraformaldehyde
*PNKP*	Polynucleotide kinase 3′-phosphatase
*PPIA*	Peptidylprolyl isomerase A
SD	Standard deviation
ST	Syncytiotrophoblast
T1D	Type-1 diabetes
*TBP*	TATA box binding protein
TNF-α	Tumor necrosis factor α
vCTs	Villous cytotrophoblasts
*WDR45L*	WD repeat domain 45B

## References

[1] Staud F., Karahoda R. (2018). Trophoblast: The central unit of fetal growth, protection and programming. Int. J. Biochem. Cell Biol..

[2] Knofler M., Haider S., Saleh L., Pollheimer J., Gamage T., James J. (2019). Human placenta and trophoblast development: Key molecular mechanisms and model systems. Cell. Mol. Life Sci. CMLS.

[3] Knofler M. (2010). Critical growth factors and signalling pathways controlling human trophoblast invasion. Int. J. Dev. Biol..

[4] Moser G., Weiss G., Sundl M., Gauster M., Siwetz M., Lang-Olip I., Huppertz B. (2017). Extravillous trophoblasts invade more than uterine arteries: Evidence for the invasion of uterine veins. Histochem. Cell Biol..

[5] Kaufmann P., Black S., Huppertz B. (2003). Endovascular trophoblast invasion: Implications for the pathogenesis of intrauterine growth retardation and preeclampsia. Biol. Reprod..

[6] Knofler M., Pollheimer J. (2013). Human placental trophoblast invasion and differentiation: A particular focus on Wnt signaling. Front. Genet..

[7] Gude N.M., Roberts C.T., Kalionis B., King R.G. (2004). Growth and function of the normal human placenta. Thrombosis Res..

[8] Hu K.-L., Chang H.-M., Zhao H.-C., Yu Y., Li R., Qiao J. (2019). Potential roles for the kisspeptin/kisspeptin receptor system in implantation and placentation. Hum. Reprod. Update.

[9] Burton G.J., Jauniaux E., Charnock-Jones D.S. (2010). The influence of the intrauterine environment on human placental development. Int. J. Dev. Biol..

[10] Majali-Martinez A., Velicky P., Pollheimer J., Knofler M., Yung H.W., Burton G.J., Tabrizi-Wizsy N.G., Lang U., Hiden U., Desoye G. (2017). Endothelin-1 down-regulates matrix metalloproteinase 14 and 15 expression in human first trimester trophoblasts via endothelin receptor type B. Hum. Reprod..

[11] Majali-Martinez A., Barth S., Lang U., Desoye G., Cervar-Zivkovic M. (2018). Temporal changes of the endothelin system in human cytotrophoblasts during the first trimester of pregnancy. Physiol. Res..

[12] Hiden U., Glitzner E., Ivanisevic M., Djelmis J., Wadsack C., Lang U., Desoye G. (2008). MT1-MMP expression in first-trimester placental tissue is upregulated in type 1 diabetes as a result of elevated insulin and tumor necrosis factor-alpha levels. Diabetes.

[13] Chang C.W., Wakeland A.K., Parast M.M. (2018). Trophoblast lineage specification, differentiation and their regulation by oxygen tension. J. Endocrinol..

[14] Burton G.J., Jauniaux E., Murray A.J. (2017). Oxygen and placental development; parallels and differences with tumour biology. Placenta.

[15] Hoch D., Gauster M., Hauguel-de Mouzon S., Desoye G. (2019). Diabesity-associated oxidative and inflammatory stress signalling in the early human placenta. Mol. Asp. Med..

[16] Pantham P., Aye I.L., Powell T.L. (2015). Inflammation in maternal obesity and gestational diabetes mellitus. Placenta.

[17] Chen C., Xu X., Yan Y. (2018). Estimated global overweight and obesity burden in pregnant women based on panel data model. PLoS ONE.

[18] Jeyabalan A. (2013). Epidemiology of preeclampsia: Impact of obesity. Nutr. Rev..

[19] Bautista-Castano I., Henriquez-Sanchez P., Aleman-Perez N., Garcia-Salvador J.J., Gonzalez-Quesada A., Garcia-Hernandez J.A., Serra-Majem L. (2013). Maternal obesity in early pregnancy and risk of adverse outcomes. PLoS ONE.

[20] Lowe W.L., Bain J.R., Nodzenski M., Reisetter A.C., Muehlbauer M.J., Stevens R.D., Ilkayeva O.R., Lowe L.P., Metzger B.E., Newgard C.B. (2017). Maternal BMI and Glycemia Impact the Fetal Metabolome. Diabetes Care.

[21] Catalano P.M., Shankar K. (2017). Obesity and pregnancy: Mechanisms of short term and long term adverse consequences for mother and child. BMJ (Clin. Res.).

[22] Velicky P., Meinhardt G., Plessl K., Vondra S., Weiss T., Haslinger P., Lendl T., Aumayr K., Mairhofer M., Zhu X. (2018). Genome amplification and cellular senescence are hallmarks of human placenta development. PLoS Genet..

[23] Lassance L., Haghiac M., Leahy P., Basu S., Minium J., Zhou J., Reider M., Catalano P.M., Hauguel-de Mouzon S. (2015). Identification of early transcriptome signatures in placenta exposed to insulin and obesity. Am. J. Obstet. Gynecol..

[24] Korgun E.T., Celik-Ozenci C., Acar N., Cayli S., Desoye G., Demir R. (2006). Location of cell cycle regulators cyclin B1, cyclin A, PCNA, Ki67 and cell cycle inhibitors p21, p27 and p57 in human first trimester placenta and deciduas. Histochem. Cell Biol..

[25] Genbacev O., McMaster M.T., Fisher S.J. (2000). A repertoire of cell cycle regulators whose expression is coordinated with human cytotrophoblast differentiation. Am. J. Pathol..

[26] Mitsuya K., Parker A.N., Liu L., Ruan J., Vissers M.C.M., Myatt L. (2017). Alterations in the placental methylome with maternal obesity and evidence for metabolic regulation. PLoS ONE.

[27] Unek G., Ozmen A., Isenlik B.S., Korgun E.T. (2017). The proliferation mechanism of normal and pathological human placentas. Histol. Histopathol..

[28] Wlodarczyk M., Nowicka G. (2019). Obesity, DNA Damage, and Development of Obesity-Related Diseases. Int. J. Mol. Sci..

[29] Lopez-Jaramillo P., Barajas J., Rueda-Quijano S.M., Lopez-Lopez C., Felix C. (2018). Obesity and Preeclampsia: Common Pathophysiological Mechanisms. Front. Physiol..

[30] Yamada Z., Kitagawa M., Takemura T., Hirokawa K. (2001). Effect of maternal age on incidences of apoptotic and proliferative cells in trophoblasts of full-term human placenta. Mol. Hum. Reprod..

[31] Lean S.C., Heazell A.E.P., Dilworth M.R., Mills T.A., Jones R.L. (2017). Placental Dysfunction Underlies Increased Risk of Fetal Growth Restriction and Stillbirth in Advanced Maternal Age Women. Sci. Rep..

[32] Varol U., Kucukzeybek Y., Alacacioglu A., Somali I., Altun Z., Aktas S., Oktay Tarhan M. (2018). BRCA genes: BRCA 1 and BRCA 2. Apoptosis.

[33] Krieger K.L., Hu W.-F., Ripperger T., Woods N.T. (2019). Functional Impacts of the BRCA1-mTORC2 Interaction in Breast Cancer. Int. J. Mol. Sci..

[34] Savage K.I., Gorski J.J., Barros E.M., Irwin G.W., Manti L., Powell A.J., Pellagatti A., Lukashchuk N., McCance D.J., McCluggage W.G. (2014). Identification of a BRCA1-mRNA splicing complex required for efficient DNA repair and maintenance of genomic stability. Mol. Cell.

[35] Deng C.X. (2006). BRCA1: Cell cycle checkpoint, genetic instability, DNA damage response and cancer evolution. Nucleic Acids Res..

[36] Wu J., Lu L.-Y., Yu X. (2010). The role of BRCA1 in DNA damage response. Protein Cell.

[37] Kastan M.B., Bartek J. (2004). Cell-cycle checkpoints and cancer. Nature.

[38] Bartek J., Lukas J. (2003). Chk1 and Chk2 kinases in checkpoint control and cancer. Cancer Cell.

[39] Ouchi T. (2006). BRCA1 phosphorylation: Biological consequences. Cancer Biol. Ther..

[40] Okada S., Ouchi T. (2003). Cell cycle differences in DNA damage-induced BRCA1 phosphorylation affect its subcellular localization. J. Biol. Chem..

[41] West R.C., McWhorter E.S., Ali A., Goetzman L.N., Russ J.E., Gonzalez-Berrios C.L., Anthony R.V., Bouma G.J., Winger Q.A. (2019). HMGA2 is regulated by LIN28 and BRCA1 in human placental cells. Biol. Reprod..

[42] West R.C., Russ J.E., Bouma G.J., Winger Q.A. (2019). BRCA1 regulates HMGA2 levels in the Swan71 trophoblast cell line. Mol. Reprod. Dev..

[43] Gauster M., Maninger S., Siwetz M., Deutsch A., El-Heliebi A., Kolb-Lenz D., Hiden U., Desoye G., Herse F., Prokesch A. (2018). Downregulation of p53 drives autophagy during human trophoblast differentiation. Cell. Mol. Life Sci. CMLS.

[44] Goldman-Wohl D., Yagel S. (2014). United we stand not dividing: The syncytiotrophoblast and cell senescence. Placenta.

[45] Henderson B.R. (2012). The BRCA1 Breast Cancer Suppressor: Regulation of Transport, Dynamics, and Function at Multiple Subcellular Locations. Scientifica.

[46] Mahmoud A.M., Macias V., Al-Alem U., Deaton R.J., Kadjaksy-Balla A., Gann P.H., Rauscher G.H. (2017). BRCA1 protein expression and subcellular localization in primary breast cancer: Automated digital microscopy analysis of tissue microarrays. PLoS ONE.

[47] Ling C., Rönn T. (2019). Epigenetics in Human Obesity and Type 2 Diabetes. Cell Metab..

[48] Nadhan R., Vaman J.V., Sengodan S.K., Hemalatha S.K., Nirmala C., Sadasivan S., Aysha P.V., Yesodharan S., Krishnapriya R.S., Amritha Krishna B.V. (2019). BRCA1 promoter hypermethylation in human placenta: A hidden link with beta-hCG expression. Carcinogenesis.

[49] Pendeloski K.P.T., Ono E., Torloni M.R., Mattar R., Daher S. (2017). Maternal obesity and inflammatory mediators: A controversial association. Am. J. Reprod. Immunol..

[50] Silva C., Nunes C., Correia-Branco A., Araújo J.R., Martel F. (2017). Insulin Exhibits an Antiproliferative and Hypertrophic Effect in First Trimester Human Extravillous Trophoblasts. Reprod. Sci..

[51] Higgins L., Greenwood S.L., Wareing M., Sibley C.P., Mills T.A. (2011). Obesity and the placenta: A consideration of nutrient exchange mechanisms in relation to aberrant fetal growth. Placenta.

[52] Scott-Drechsel D.E., Rugonyi S., Marks D.L., Thornburg K.L., Hinds M.T. (2013). Hyperglycemia slows embryonic growth and suppresses cell cycle via cyclin D1 and p21. Diabetes.

[53] Bordeleau L., Lipscombe L., Lubinski J., Ghadirian P., Foulkes W.D., Neuhausen S., Ainsworth P., Pollak M., Sun P., Narod S.A. (2011). Diabetes and breast cancer among women with BRCA1 and BRCA2 mutations. Cancer.

[54] Aoki M., Murase T. (2019). Obesity-associated insulin resistance adversely affects skin function. PLoS ONE.

[55] Jauniaux E., Watson A.L., Hempstock J., Bao Y.P., Skepper J.N., Burton G.J. (2000). Onset of maternal arterial blood flow and placental oxidative stress. A possible factor in human early pregnancy failure. Am. J. Pathol..

[56] Li L., Cohen M., Wu J., Sow M.H., Nikolic B., Bischof P., Irminger-Finger I. (2007). Identification of BARD1 splice-isoforms involved in human trophoblast invasion. Int. J. Biochem. Cell Biol..

[57] Benowitz N.L., Bernert J.T., Caraballo R.S., Holiday D.B., Wang J. (2009). Optimal serum cotinine levels for distinguishing cigarette smokers and nonsmokers within different racial/ethnic groups in the United States between 1999 and 2004. Am. J. Epidemiol..

[58] Strutz J., Cvitic S., Hackl H., Kashofer K., Appel H.M., Thüringer A., Desoye G., Koolwijk P., Hiden U. (2018). Gestational diabetes alters microRNA signatures in human feto-placental endothelial cells depending on fetal sex. Clin. Sci..

[59] Maksimovic J., Gordon L., Oshlack A. (2012). SWAN: Subset-quantile within array normalization for illumina infinium HumanMethylation450 BeadChips. Genome Biol..

[60] Phipson B., Maksimovic J., Oshlack A. (2016). missMethyl: An R package for analyzing data from Illumina’s HumanMethylation450 platform. Bioinformatics.

[61] The R Development Core Team (2018). R: A Language and Environment for Statistical Computing.

[62] Wickham H. (2016). Ggplot2: Elegant Graphics for Data Analysis.

[63] Baptiste A. (2017). gridExtra: Miscellaneous Functions for “Grid” Graphics. R Package Version 2.3.

[64] Plotly Technologies, Inc (2015). Collaborative Data Science.

