# Supplementary material for: Maternal Obesity Alters Placental Cell Cycle Regulators in the First Trimester of Human Pregnancy: New Insights for BRCA1
Source: Int J Mol Sci. 2020 Jan 11;21(2):468. doi: 10.3390/ijms21020468 (PMC7014057; doi:10.3390/ijms21020468)
Supplement: Supplementary file 1 [file ijms-21-00468-s001.pdf]

Supplementary Material and Methods

Blood Collection and Cotinine Determination

Venous blood was collected after overnight fasting in S-Monovette collection tubes (Sarstedt, Nümbrecht, Germany) and centrifuged at 2000xg for 10 minutes at room temperature. After aliquoting, the serum fraction was immediately frozen at -80°C.

Serum cotinine levels were measured with a solid phase competitive cotinine Enzyme-linked Immunosorbent Assay (ELISA, Abnova, Taipei, China) according to manufacturer's guidelines. The cut-off defining smoker was set to cotinine levels ≥ 0.03nmol/L [57]. Analytical assay sensitivity was 1ng/mL with cross-reactivity for nicotine (<1%), nicotinamide (<1%) and nicotinic acid (<1%).

Measurement of Secreted hCG

Supernatant was collected for all treatments, centrifuged at 1800x*g* for 10 minutes and stored at -20°C until immunoassay analysis. Human chorionic gonadotropin (hCG) levels were determined using IMMULITE 1000 Systems Immunoassay (Siemens, Munich, Germany). Results were normalized to total protein content in each well.

Supplementary Tables and Figures

Supplementary Table S1. Genes upregulated by maternal obesity in first trimester placental tissue.

Coefficients					
	Unstandardized Coefficients		Standardized Coefficients	t	Sig.
**Dependent Variable**	**B**	**Std. Error**	**Beta**		
ZNF350	-0.367	0.045	-0.452	-8.140	0.015
ERCC5	0.744	0.092	0.945	8.123	0.015
CREBBP	-0.326	0.048	-1.040	-6.804	0.021
SLC35G6	-0.499	0.079	-1.045	-6.359	0.024
LIG4	-0.720	0.122	-0.890	-5.913	0.027
BRCA1	-1.515	0.585	-0.715	-2.590	0.029
POLB	-0.802	0.156	-0.765	-5.127	0.036
FANCL	-0.654	0.143	-0.935	-4.581	0.044
ERCC1	-1.058	0.240	-1.041	-4.418	0.048
PNKP	-0.743	0.173	-1.006	-4.295	0.050
MAX	-0.514	0.128	-1.015	-4.010	0.057
GTF2H1	-0.659	0.175	-1.021	-3.770	0.064
CSNK2A2	-0.596	0.161	-0.962	-3.703	0.066
SUMO1	-0.655	0.181	-0.764	-3.618	0.069
AKT2	-1.046	0.296	-1.013	-3.538	0.071
POLL	-0.866	0.250	-0.959	-3.470	0.074
RAD23B	-1.685	0.520	-0.994	-3.243	0.083
POLR2E	-0.837	0.263	-0.967	-3.185	0.086
RBX1	-1.524	0.499	-0.992	-3.054	0.093

Multivariate linear regression model with BMI as exposure variable, correcting for maternal age.

Supplementary Table S2. Proteins upregulated by maternal obesity in first trimester placental tissue.

Coefficients					
	Unstandardized Coefficients		Standardized Coefficients	t	Sig.
Dependent Variable	B	Std. Error	Beta		
**RAD52 Ab104**	39.565	0.214	1.000	185.074	0.000
**HDAC1 Ab421**	23.151	2.853	0.951	8.114	0.000
**Cyclin E1 Ab77**	11.900	1.960	0.916	6.071	0.001
**BRCA1 Ab1423**	16.374	3.353	0.878	4.883	0.002
**Myc Ab358**	5.800	1.268	0.849	4.575	0.003
**14-3-3 zeta/delta Ab232**	5.678	1.249	0.857	4.547	0.003
**P90RSK Ab359/363**	8.611	2.010	0.850	4.283	0.004
**CDC25C Ab216**	8.937	2.091	0.830	4.274	0.004
**Chk2 Ab68**	3.440	0.857	0.834	4.013	0.005
**p95 Ab343**	2.067	0.516	0.834	4.003	0.005
**ATRIP Ab68/72**	5.807	1.480	0.816	3.925	0.006
**HDAC6 Ab22**	2.693	0.699	0.824	3.853	0.006
**HDAC3 Ab424**	1.517	0.403	0.818	3.761	0.007
**HDAC8 Ab39**	0.958	0.255	0.817	3.752	0.007
**MDM2 Ab166**	12.170	3.307	0.811	3.680	0.008
**BRCA1 Phospho-Ser1423**	2.289	0.636	0.747	3.598	0.009
**PP2A Ab307**	3.204	0.946	0.786	3.385	0.012
**Chk1 Ab286**	1.672	0.497	0.770	3.363	0.012
**Cyclin D1 Ab286**	3.154	0.944	0.780	3.341	0.012
**ABL1 Ab204**	15.187	4.777	0.768	3.179	0.016
**BRCA1 Ab1524**	1.405	0.472	0.731	2.976	0.021
**Smad2 3 Ab8**	0.599	0.214	0.723	2.795	0.027
**E2F1 Ab433**	3.691	1.324	0.725	2.788	0.027
**Cyclin D1 Phospho-Thr286**	2.885	1.058	0.717	2.727	0.029
**Smad3 Phospho-Ser213**	1.043	0.404	0.696	2.585	0.036
**TGFBR2 Ab250**	1.692	0.664	0.693	2.546	0.038
**TOP2A Ab1106**	1.169	0.514	0.649	2.272	0.057
**p53 Ab20**	1.187	0.531	0.639	2.233	0.061
**Smad2 3 Phospho-Thr8**	0.574	0.257	0.642	2.233	0.061
**HDAC1 Phospho-Ser421**	1.077	0.487	0.637	2.212	0.063
**DNA PK Ab2638**	1.230	0.557	0.635	2.209	0.063
**PLK1 Ab210**	1.510	0.696	0.617	2.169	0.067
**Cyclin A1 C-term**	2.420	1.211	0.601	1.999	0.086
**p53 Ab18**	0.527	0.270	0.583	1.951	0.092
**Cyclin E2 Ab392**	0.610	0.315	0.586	1.934	0.094

Multivariate linear regression model with BMI as exposure variable, correcting for maternal age.

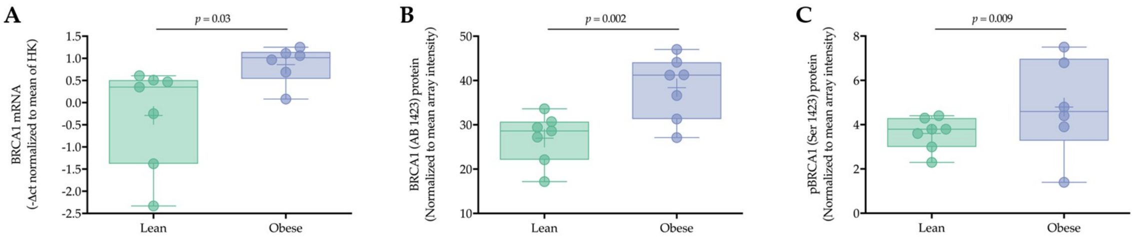

Supplementary Figure S1. Placental BRCA1 is upregulated by maternal obesity. BRCA1 expression and BRCA1 and p(Ser1423)-BRCA1 protein levels were determined by PCR panel (**A**) and Protein array (**B** and **C**), respectively, in first trimester placental tissue (gestational week 7) from lean (n = 7) and obese (n = 6) women. PCR Panel data are normalized to the mean of two housekeeping genes (HPRT1 and TBP) and Protein array data were normalized on the median intensity value of all antibodies on each array. Results are presented as mean ± SD and analyzed using a multivariate linear regression model adjusting for maternal age.

Supplementary Table S3. DNA methylation of *BRCA1* gene in lean (gestational week 6^{+0}-11^{+6}, n = 15) vs. obese (gestational week 5^{+0}-11^{+2}, n = 15) first trimester placental tissue.

	**Lean**			**Obese**		
	Mean	SD	n	Mean	SD	n
cg06706355	0.401	0.043	15	0.4	0.025	15
cg20334877	0.679	0.034	15	0.695	0.033	15
cg02697717	0.439	0.029	15	0.456	0.025	15
cg02190078	0.493	0.029	15	0.5	0.029	15
cg16490202	0.09	0.009	15	0.082	0.007	15
cg05514735	0.179	0.026	15	0.174	0.033	15
cg25676905	0.112	0.043	15	0.106	0.022	15
cg11808250	0.034	0.007	15	0.033	0.006	15
cg09575269	0.041	0.006	15	0.038	0.004	15
cg00902415	0.057	0.009	15	0.057	0.007	15
cg14995433	0.055	0.006	15	0.055	0.006	15
cg18208654	0.035	0.012	15	0.036	0.005	15
cg16450103	0.043	0.006	15	0.04	0.006	15
cg18831773	0.558	0.018	15	0.559	0.015	15
cg08041014	0.481	0.036	15	0.485	0.034	15
cg01178231	0.88	0.027	15	0.895	0.017	15
cg24818472	0.919	0.019	15	0.912	0.015	15
cg20071259	0.885	0.02	15	0.891	0.013	15
cg17091024	0.737	0.027	15	0.743	0.026	15
cg22325646	0.21	0.027	15	0.213	0.039	15
cg25504443	0.127	0.025	15	0.12	0.027	15
cg16879222	0.033	0.004	15	0.03	0.004	15
cg16246366	0.041	0.006	15	0.042	0.007	15
cg17225407	0.157	0.071	15	0.147	0.066	15
cg12943674	0.074	0.008	15	0.074	0.005	15
cg26524541	0.077	0.007	15	0.076	0.007	15
cg10162691	0.05	0.012	15	0.05	0.009	15
cg05270634	0.048	0.008	15	0.046	0.009	15
cg07591186	0.553	0.052	15	0.558	0.033	15
cg08732879	0.42	0.05	15	0.409	0.025	15
cg18068798	0.657	0.038	15	0.663	0.028	15
cg25061307	0.73	0.05	15	0.726	0.033	15
cg14648161	0.387	0.032	15	0.378	0.037	15
cg09967497	0.415	0.059	15	0.409	0.04	15
cg21983245	0.82	0.028	15	0.832	0.027	15
cg05530195	0.446	0.047	15	0.439	0.037	15
cg23693293	0.538	0.055	15	0.529	0.044	15
cg01879757	0.683	0.09	15	0.709	0.078	15
cg16029534	0.876	0.015	15	0.875	0.019	15
cg16919093	0.901	0.034	15	0.914	0.019	15
cg18830083	0.728	0.045	15	0.74	0.04	15
cg25031275	0.68	0.033	15	0.687	0.022	15
cg23084950	0.856	0.031	15	0.863	0.015	15
cg16550131	0.489	0.044	15	0.472	0.027	15
cg05758696	0.801	0.018	15	0.81	0.019	15
cg17526501	0.904	0.019	15	0.918	0.01	15
cg09648631	0.878	0.027	15	0.867	0.021	15
cg07054526	0.896	0.02	15	0.901	0.017	15
cg14048487	0.748	0.037	15	0.742	0.034	15
cg19531713	0.1	0.01	15	0.098	0.011	15
cg19088651	0.041	0.012	15	0.037	0.008	15
cg08386886	0.187	0.018	15	0.184	0.016	15
cg08993267	0.044	0.006	15	0.041	0.005	15
cg24806953	0.035	0.007	15	0.029	0.008	15
cg20187250	0.03	0.005	15	0.029	0.005	15
cg15419295	0.046	0.008	15	0.043	0.013	15
cg16963062	0.029	0.008	15	0.027	0.006	15
cg16630982	0.028	0.007	15	0.028	0.006	15
cg21253966	0.034	0.011	15	0.036	0.008	15
cg04110421	0.04	0.013	15	0.036	0.006	15
cg04658354	0.033	0.006	15	0.029	0.004	15
cg17301289	0.042	0.009	15	0.041	0.006	15
cg09441966	0.037	0.009	15	0.035	0.011	15
cg26891576	0.197	0.031	15	0.182	0.026	15
cg20760063	0.049	0.005	15	0.05	0.005	15
cg10125569	0.04	0.005	15	0.035	0.005	15
cg01587050	0.032	0.006	15	0.028	0.004	15
cg10893007	0.031	0.006	15	0.029	0.003	15
cg12182452	0.038	0.008	15	0.038	0.007	15
cg09831010	0.04	0.005	15	0.037	0.006	15
cg25067162	0.073	0.021	15	0.074	0.017	15
cg26276233	0.115	0.039	15	0.112	0.028	15
cg06001716	0.095	0.059	15	0.087	0.022	15
cg02286533	0.113	0.055	15	0.112	0.036	15
cg14947218	0.126	0.057	15	0.126	0.037	15
cg16006004	0.095	0.051	15	0.095	0.033	15
cg18372208	0.146	0.053	15	0.148	0.04	15
cg25288140	0.19	0.076	15	0.197	0.059	15
cg15065591	0.178	0.079	15	0.181	0.058	15
cg11529738	0.144	0.05	15	0.152	0.034	15
cg24900425	0.138	0.057	15	0.139	0.041	15
cg06973652	0.102	0.035	15	0.106	0.028	15
cg20185525	0.42	0.018	15	0.424	0.024	15
cg26879546	0.487	0.071	15	0.494	0.083	15
cg10200559	0.612	0.04	15	0.615	0.039	15
cg14687471	0.858	0.023	15	0.862	0.02	15

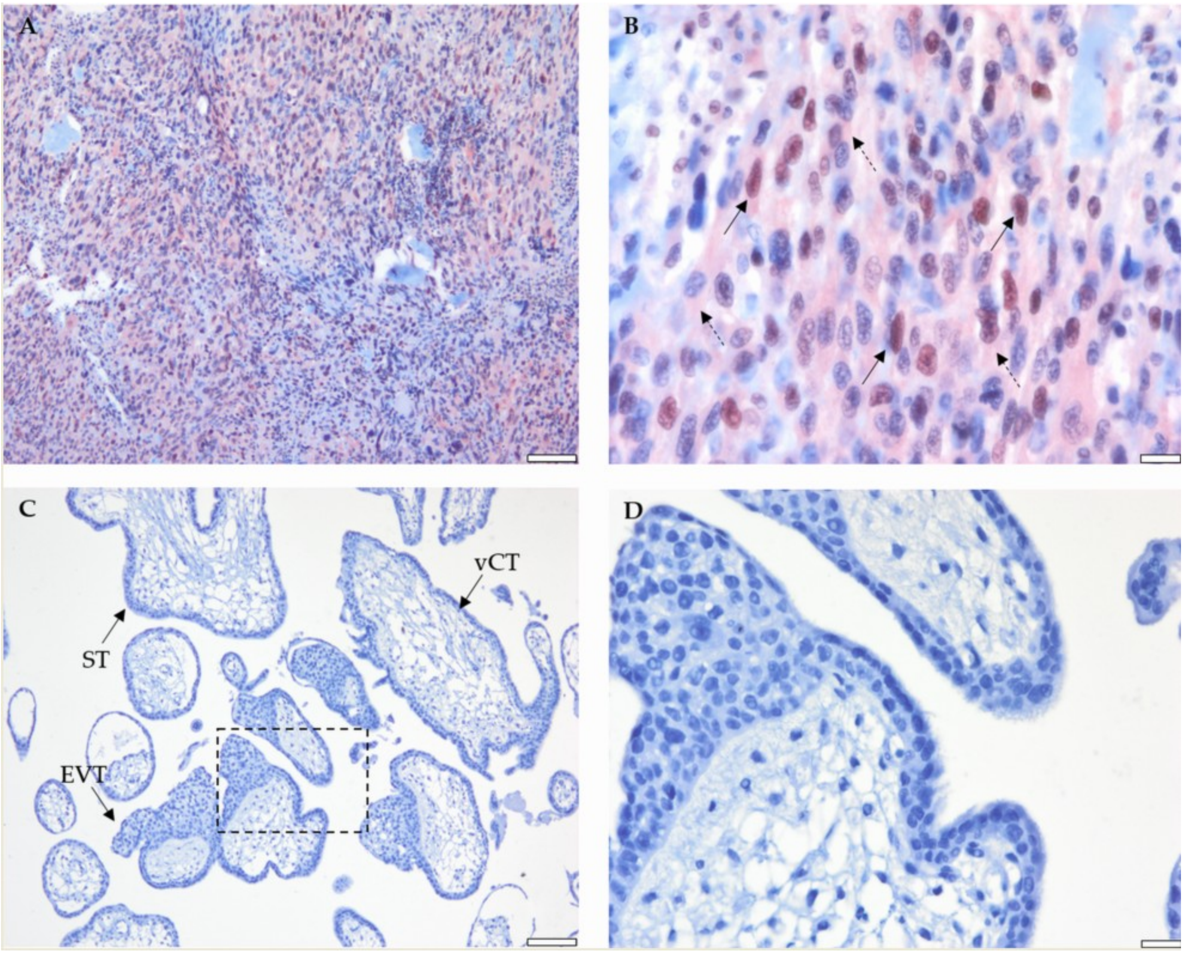

Supplementary Figure S2. Positive and negative controls for BRCA1 immunohistochemistry of placental tissue. An ovarian cancer specimen was used as positive control (**A** and **B**), showing BRCA1 nuclear (**B**, arrows) and cytosolic (**B**, dotted arrows). IgG isotype negative control at the same primary antibody concentration showed no staining (**C** and **D**). Scale bar: 100µm (**A** and **C**) or 20µm (**B** and **D**). Dotted frame in **C** indicates the field shown with a higher magnification in **D**.

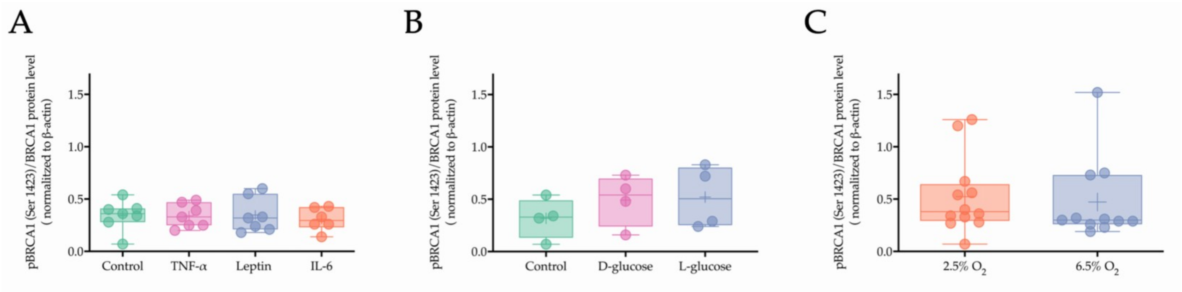

Supplementary Figure S3. p$^{(Ser1423)}$-BRCA1/BRCA1 ratio is not regulated by short term exposure to obesity-associated inflammation, hyperglycemia or oxygen tension in early pregnancy. First trimester placental chorionic villous explants from different placental tissues (*n* = 4-11) were cultured at 2.5% O_2 with (**A**) TNF-*α* (50ng/mL), leptin (100ng/mL) and IL-6 (100ng/mL) or (**B**) D-glucose (25nM) and L-glucose (25nM, osmotic control) for 48 hours in triplicates. Explants (*n* = 10-12) were also cultured at 6.5% O_2 (**C**). p$^{(Ser1423)}$-BRCA1/BRCA1 protein ratio was calculated based on densitometric analysis from immunoblotting. Results are presented as mean ± SD. Statistical analysis included Mann Whitney test or Friedman's test followed by Dunn's post hoc analysis.

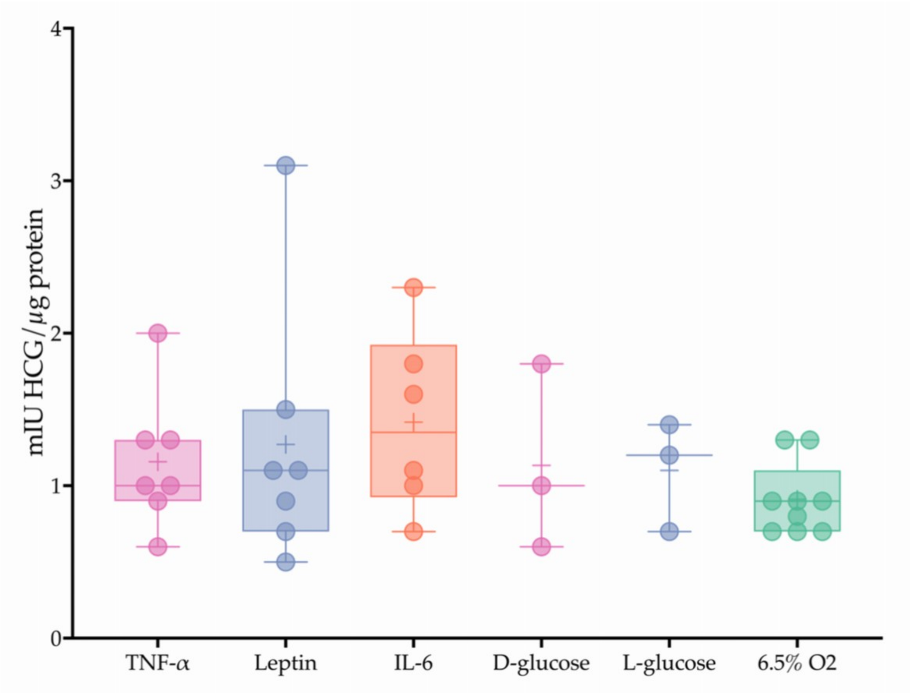

Supplementary Figure S4. Human chorionic gonadotropin (hCG) levels in culture supernatants of placental chorionic villi explants remained stable after exposure to TNF-α, leptin, IL-6, D-glucose, L-glucose at 2.5% oxygen. Increasing the oxygen tension to 6.5% did not change secreted hCG. Data is shown as mlU hCG normalized on total protein content in the respective well. Results are expressed as mean ± SD. Statistical analysis was performed using One-way ANOVA.
